# Effect of the core bone engaged length on the BASHTI fixation strength, an in-vitro study on bovine tendons using identical-density surrogate bones

**DOI:** 10.1186/s12891-023-06311-2

**Published:** 2023-03-25

**Authors:** Hadi Moeinnia, Amir Nourani, Mahdi Mohseni, Amirhossein Borjali, Narges Ghias, Hossein Korani, Mahmoud Chizari

**Affiliations:** 1 Department of Mechanical Engineering, Sharif University of Technology, Tehran, Iran; 2 School of Engineering, Physics and Computer Sciences, University of Hertfordshire, Hatfield, UK

**Keywords:** ACL reconstruction, BASHTI technique, Fixation strength, Bone plug, Knee biomechanics, Orthopaedic biomechanics

## Abstract

**Background:**

BASHTI is an implant-less anterior cruciate ligament (ACL) reconstruction technique, which resolves the problems caused by implants such as interference screws. This study aims to investigate the effect of the drill bit and tendon’s diameter on the Core Bone Engaged Length (CBEL) and the fixation strength. CBEL is the length of core bone which has a full engagement with both tunnel and graft at the same time.

**Methods:**

60 in-vitro tests were conducted for 6, 7, 8, and 9 mm tendon sizes with a 10 mm bone tunnel. In this study bovine tendons and dummy bone blocks were used to model the fixation. Drill bits were used to extract the core bone for securing the auto-graft. A three-stage tensile test including a force-controlled cyclical preloading of 10–50 N with a frequency of 0.1 Hz for 10 cycles, followed by the main force-controlled cyclical loading of 50–200 N with a frequency of 0.5 Hz for 150 cycles, and immediately a displacement-controlled single cycle pull-out load with a rate of 20 mm/min were carried out to discover the fixation strength of each sample.

**Results:**

The 6 mm group had the greatest CBEL. However, all cases in this group failed in loadings below 200 N, which is the minimum required strength after ACL reconstruction. The fixation strength of cases with more than 200 N fixation strength for 7, 8, and 9 mm tendon diameters were 275 ± 42, 330 ± 110, and 348 ± 93 N, respectively, showing insignificant difference between groups (*P-value* = 0.45). Nevertheless, CBELs for these groups were 16.6 ± 3.4, 9.6 ± 2.4, and 11.7 ± 3.8 mm, respectively, implying a significant increase in CBEL in the 7 mm group than that for 8 and 9 mm groups (*P-value* = 0.002 and 0.049, respectively).

**Conclusion:**

Results showed that CBEL could assess the quality of BASHTI technique. However, CBEL was an inverse function of tendon compression, so it was not an independent parameter to determine BASHTI strength. Also, the CBEL of 7 mm group which fulfilled the 200 N threshold was higher than that of 8 and 9 mm groups, so its healing process speed may be higher, which is recommended for a future study in this field.

## Background

The anterior cruciate ligament (ACL) has an essential role in stabilizing the rotational movements of the knee [1]. ACL injuries, including partial or complete ligament tear, might occur under extreme sports activities, mostly as a result of a non-contact injury [2]. In a complete ACL tear, due to a lack of self-healing process, surgical reconstruction is required [3]. Using an interference screw is the most frequent fixation method in these surgeries [4, 5]. Nevertheless, this fixation method can be criticized for requiring expensive equipment and problems such as bone tunnel enlargement [6], inflammatory responses [7], and tendon rotation [8]. Consequently, implant-free techniques have been presented as a substitute for conventional techniques to reconstruct the ACL. The best-known implant-free technique is the press-fit method which uses bone plugs at the two ends of the patellar graft tissue for fixation [9]. This technique, however, is criticized due to problems such as the constrained length of the auto-graft [10].

BASHTI technique is an implant-free fixation method that uses a hamstring tendon and a core bone harvested during bone tunneling. The fixation strength of BASHTI and interference screw techniques is compared in both artificial bone and bovine models, indicating no significant differences between these two methods [11, 12]. Also, it is shown that increasing the geometric parameters (e.g., tendon and core bone diameter) to a critical value would increase BASHTI fixation strength [13–15]. In addition, the sheathed core bone can be inserted into the tunnel with less hammer impact force and reduce the risk of cracking on the core bone during the insertion process and increase the fixation strength [16, 17]. Moreover, it is shown that BASHTI fixation strength is significantly affected by the bone density and core bone insertion frequency (i.e., hammer strike rate during the insertion process) [18–20].

In the insertion process of a core bone into a bony tunnel using a hammer, only a portion of its length would be inserted into the tunnel. This is mainly because of the local fracture of the core bone due to the hammer impacts. The inserted length of the core bone is called the core bone engaged length (CBEL). It is believed that as the contact area between the core bone and the bone tunnel increases, the healing process speed, as well as friction and impact forces, would increase [21]. So, it is important to investigate the effect of involved parameters on the amount of CBEL. This study aims to examine the effect of CBEL on the BASHTI fixation strength and discusses its relationship with the cannulated drill bit and tendon diameter.

## Methods

**Fig. 1:**
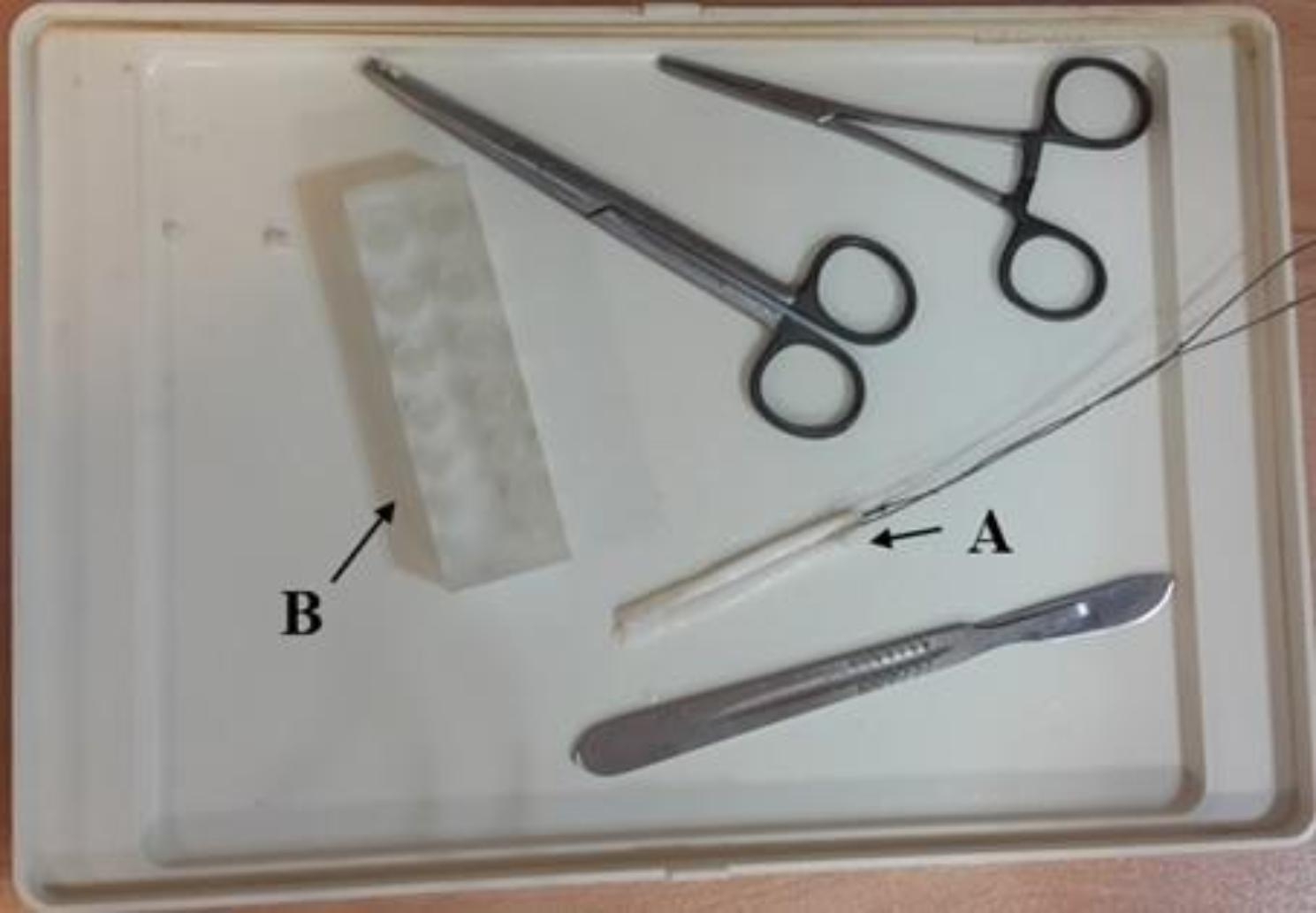
A typical double-strand tendon prepared for the test (A), tendon trimming equipment including surgical blade, gauge, and forceps (B)

**Fig. 2:**
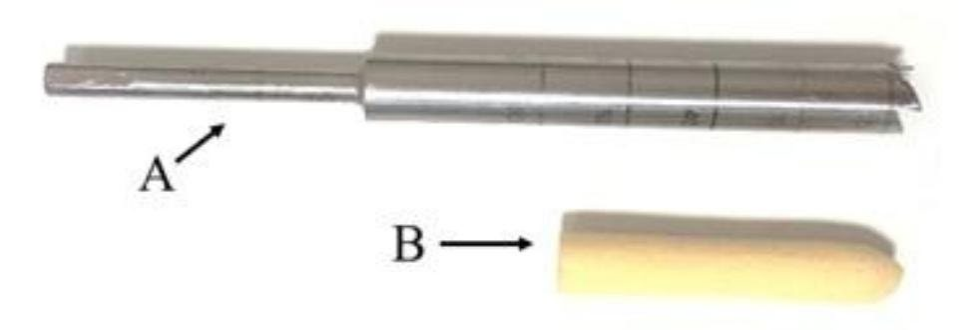
A BASHTI’s cannulated drill bit (A) and its extracted core bone (B)

This experimental in-vitro study uses digital tendons harvested freshly from bovine hoofs. These hoofs were bought freshly from a licensed butchery. For consistency of the results, the hoofs were selected from the same breed and close age bovines. It has been already confirmed that the property of the grafts made from bovine digital extensor tendons is similar to human hamstring tendon [22]. These tendons were precisely trimmed to 6, 7, 8, and 9 mm diameters using laboratory sizing equipment (Fig. 1). Tendon trimming was done using a precision cutter by laboratory operator. The tendon was placed on a cutting board and then trimmed to the desired size. To verify the tendon size, the tendon is passed through the related gauge’s hole (Fig. 1). If the tendon is perfectly fitted through the hole, the corresponding bore size is considered as the size of the tendon; otherwise, the sample is failed and a new one must be prepared. The gauge had a range of 6–12 mm diameter bore sizes with a 0.5 mm interval. Accordingly, the measurement accuracy would be 0.5 mm. Tendons were stored at -20 °C for less than 48 h so their mechanical properties did not change [23]. Moreover, the Sawbones artificial bone blocks (Pacific Research Laboratories, Malmo, Sweden**)** with the same size and the same density of 320 kg/m^3^ – which is proofed to have similar mechanical properties with femoral/tibial cancellous bones - were used as an alternative for the cancellous bone of a young human [20, 24]. In addition, BASHTI’s cannulated drill bit was used to extract a ~ 30 mm core bone safely from the Sawbones (Fig. 2). These drill bits are used to extract the desired cylindrical core bones from the cancellous bone site while drilling the fixation bone tunnel. The outer diameter of the drill bits is set to 10 mm, which is appropriate for ACL reconstruction surgeries [25]. The inner diameter was made in different sizes to be suitable for the fixation of 6, 7, 8, and 9 mm tendon grafts [26]. Table 1 shows the drill bits and tendon diameters used in this study.

**Table 1:** Dimensions of the tendon, core bones, and inner size of drill bits

Tunnel (mm)	Tendon (mm)	Core bone (mm)	Drill bit inner diameter (mm)
10	6	9.5*	9.9
		9	9.4
		8.5	8.9
	7	9.5*	9.9
		9	9.4
		8.5	8.9
	8	9*	9.4
		8.5	8.9
		8	8.4
	9	8.5*	8.9
		8	8.4
		7.5	7.9

* Maximum core bone sizes respect to each tendon diameter

**Fig. 3:**
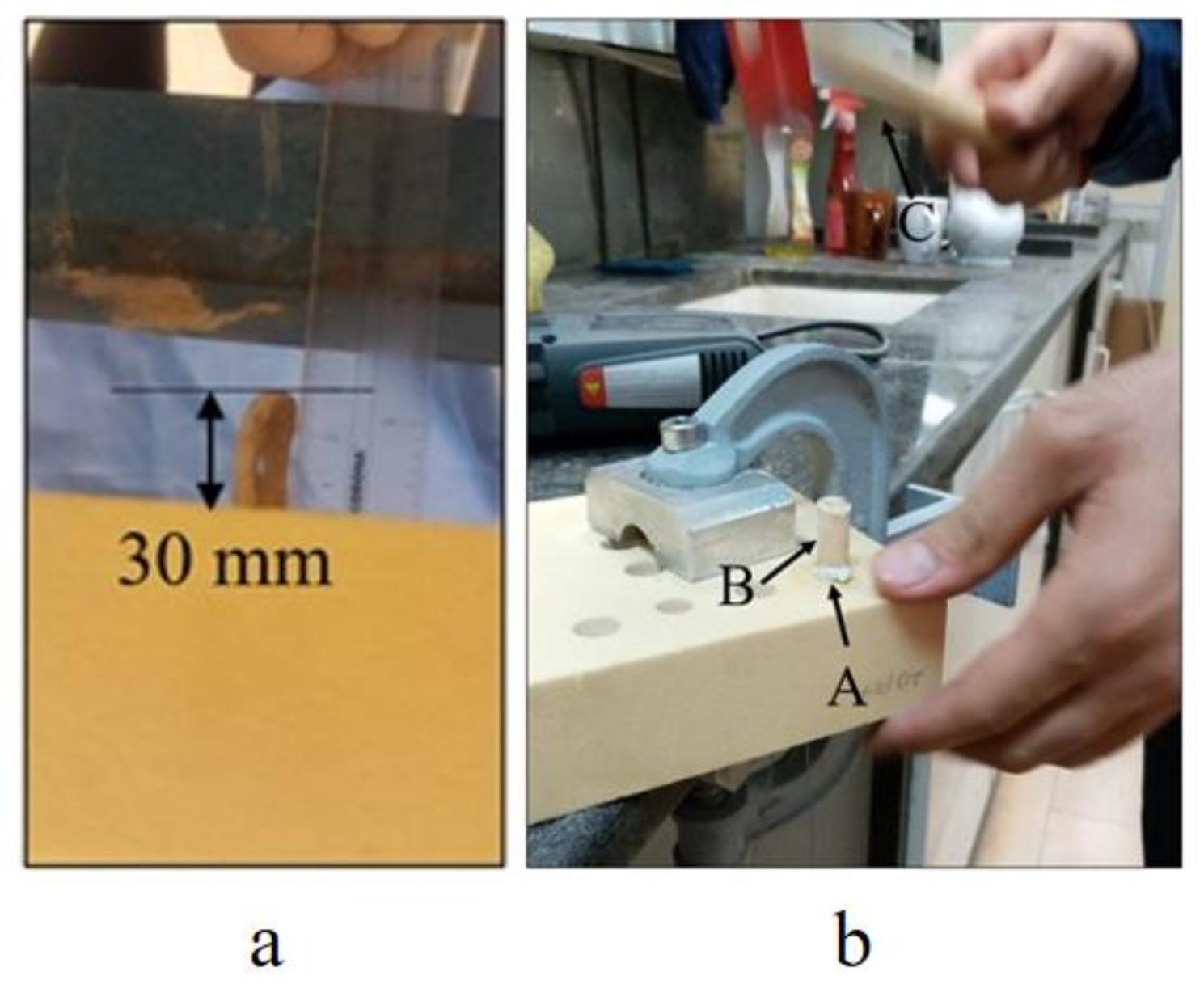
(a) The 30 mm gauge length of the tendon graft left free outside of the tunnel, (b) Core bone insertion process: securing the tendon (A) into the tunnel with a core bone (B) using a hand-powered (manual) hammer (C)

To secure the tendon graft into the tunnel, the tendon was doubled and passed through the tunnel, maintaining a gauge length of 30 mm tendon graft left free outside of the tunnel (Fig. 3a). This gauge length corresponded to the length of natural intact ACL [23]. Following the tendon insertion, the core bone was inserted into the tunnel. The insertion process was conducted using a hand-powered hammer by applying a frequency lower than 300 beats per minute on the top of the core bone in line with its central axis (Fig. 3b) [19]. The tendon was kept moist during the insertion process. The results of a power analysis (considering alpha = 0.05 and effect size = 0.5) indicated that the minimum number of repetitions required for this study was five (statistical power > 0.99). To check the repeatability of the results, therefore, five of each sample was built. Finaly, 60 different in-vitro BASHTI ACL fixation samples (4 different tendon diameters × 3 different dore bone diameters for each tendon diameter × 5 test repeats) were built for this study.

**Fig. 4:**
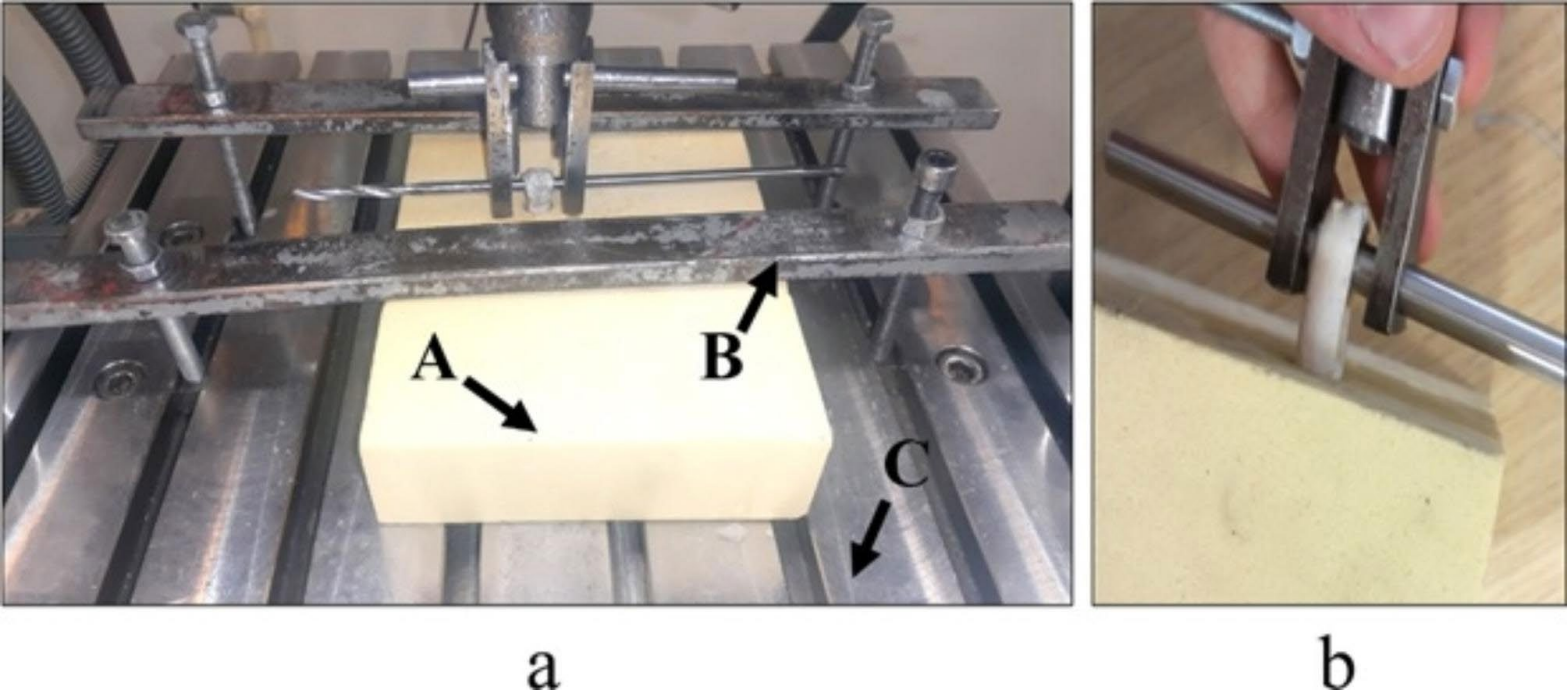
a. Testing setup: Sawbones block (A) fixed using a custom-made holder (B) on the servo-hydraulic machine platform (C), b. The double-strand portion of the tendon was hung on the gripper

**Fig. 5:**
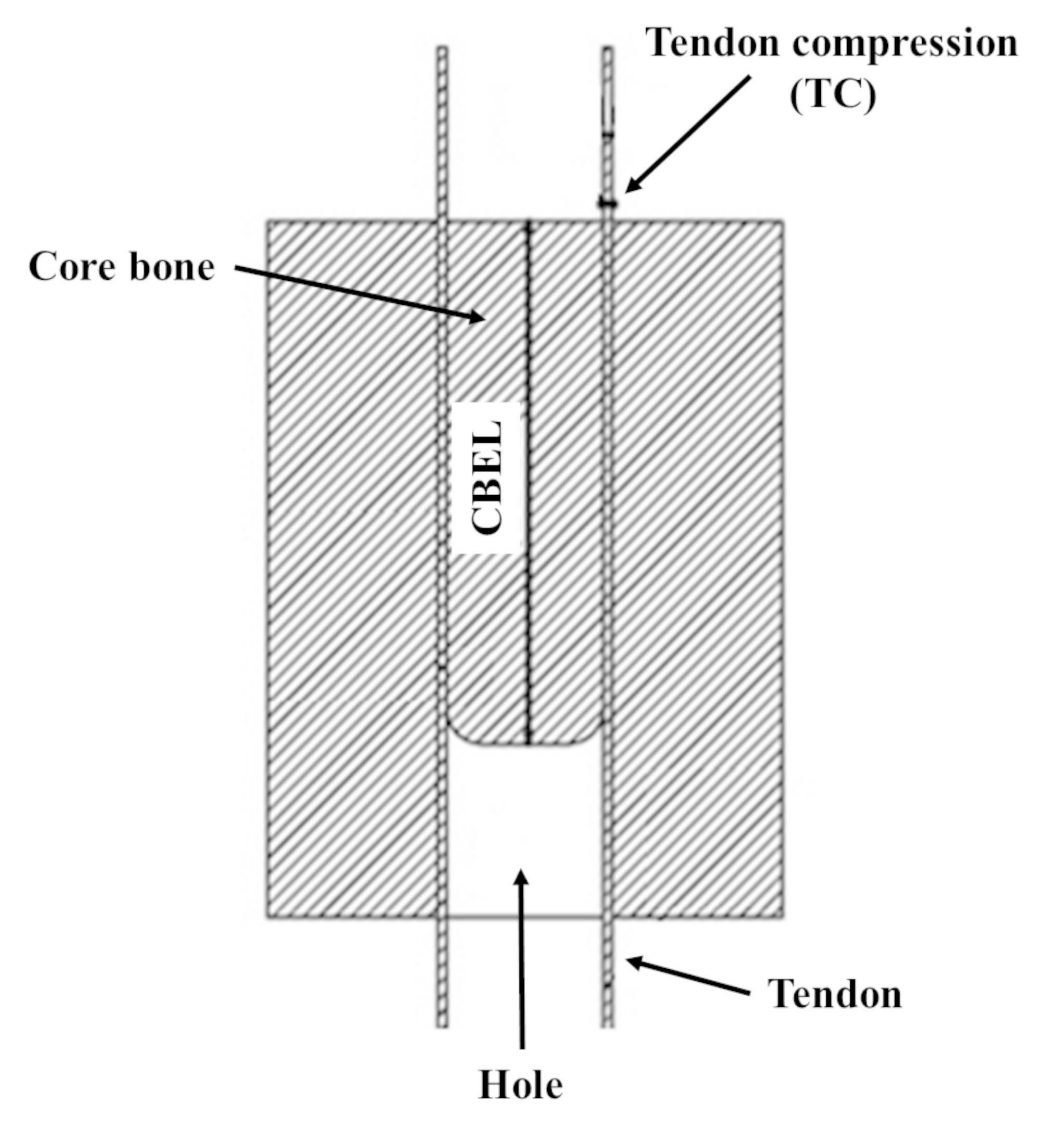
Two parameters of tendon compression and CBEL

Shortly after the insertion process, the specimen was mounted into a servo-hydraulic machine (Amsler HCT 25–400; Zwick/Roell AG, Germany) to assess the mechanical properties of the fixation. The Sawbones block was mounted on the testing machine using a custom-made rig (Fig. 4a). Also, a custom-made hanger was used to hold the specimen (Fig. 4b). Initially, a cyclical preconditioning load of 10–50 N with a frequency of 0.1 Hz for 10 cycles was applied to the specimen. The preconditioning load was applied to eliminate the tendon graft’s loose length and prepare it for the next loading steps [27]. Immediately after that, the main force-controlled cyclical loading was applied to the specimen. This was set to 50–200 N with a frequency of 0.5 Hz for 150 cycles. This step was planned to simulate the ACL passive flexion-extension loading forces applied to the knee during the early rehabilitation process of a reconstructed graft [28, 29]. Following the main cyclical test, a displacement-controlled single cycle pull-out load with a rate of 20 mm/min was applied to the specimens to measure the failure strength of the fixation [30]. Moreover, after the failure of each sample, the CBEL was measured for each sample by measuring the size of the final pulled-out core bone (Fig. 5). The fixation failure was monitored, either considering an elongation of more than 10 mm or a visible tendon rupture [14]. To simplify the fixation geometrical parameters (i.e. tendon, core bone, and tunnel diameters), the tendon compression (TC) was defined using Eq. (1) [13].

1 $${\rm{TC}} = \frac{{{{\rm{S}}_{{\rm{tendon}}}} + {{\rm{S}}_{{\rm{core}}}} - {{\rm{S}}_{{\rm{tunnel}}}}}}{{{{\rm{S}}_{{\rm{tendon}}}}}}$$

Where TC is a dimensionless parameter representing the tendon compression. S_tendon_, S_core,_ and S_tunnel_ are the cross-section areas of the tendon, core bone, and tunnel, respectively (Fig. 5).

A Student’s t distribution was used to calculate the 95% Confidence Interval (CI) of the results. The ANOVA method was used to analyze if the difference between the results of different tendon groups were significant. A *P-value* less than 0.05 was considered to be statistically significant.

## Results

**Fig. 6:**
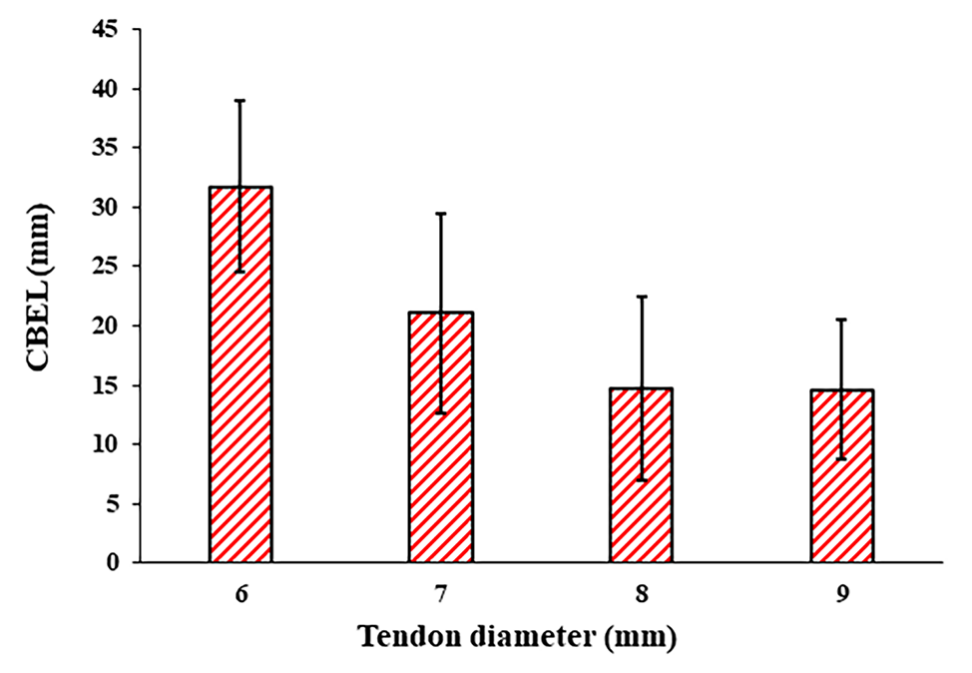
Relation between CBEL and Tendon diameter. Error bars shown at 95% CI

**Fig. 7:**
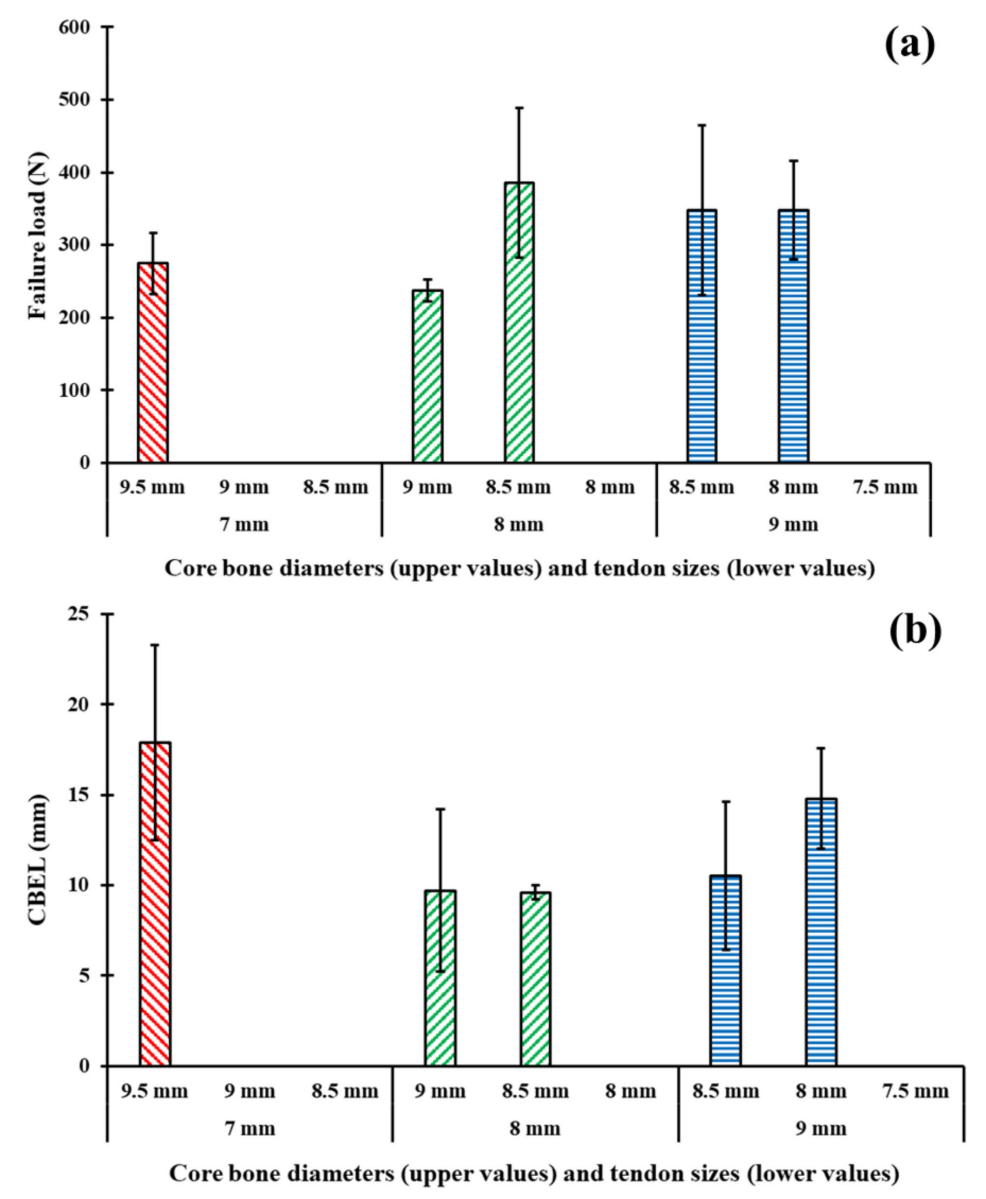
(a) Fixations strength and (b) CBEF of samples with failure loads of more than 200 N as a function of core bone diameter for different tendon size groups. Error bars shown at ± 95% CI. Groups without any bars didn’t have any samples with more than 200 N failure loads

According to the outcome obtained in current study, the tendon size had a significant effect on the CBEL, but it did not affect the fixation strength of the samples significantly. The average CBELs of the 6, 7, 8, and 9 mm tendon diameter groups were 31.7 ± 7.2, 21.1 ± 8.4, 14.7 ± 7.8, and 14.6 ± 5.9 mm (95% CI) respectively (Fig. 6). Previous studies on postoperative ACL reconstruction have shown that a load with a magnitude of at least 200 N would be applied to the fixation during a rehabilitation process [31]. Therefore, the value of 200 N was defined as the threshold to quantify CBEL. With the 6 mm tendon group, none of the samples could endure more than 200 N loadings. As a result, the 6 mm tendon was not considered for further development. The average fixation strength of samples enduring more than 200 N failure load with tendon diameters of 7, 8, and 9 mm were 275 ± 42 N, 330 ± 110 N, and 348 ± 93 N (95% CI), respectively (Fig. 7a). So, no significant difference was observed between fixation strengths (*P-value* = 0.45). On the other hand, the CBELs of these samples were 16.6 ± 3.4 mm, 9.6 ± 2.4 mm, and 11.7 ± 3.8 mm, respectively. Thus, the CBEL of the 7 mm tendon group was significantly more than that of 8 and 9 mm tendon groups (*P-value* = 0.002 and 0.049, respectively) (Fig. 7b).

**Fig. 8:**
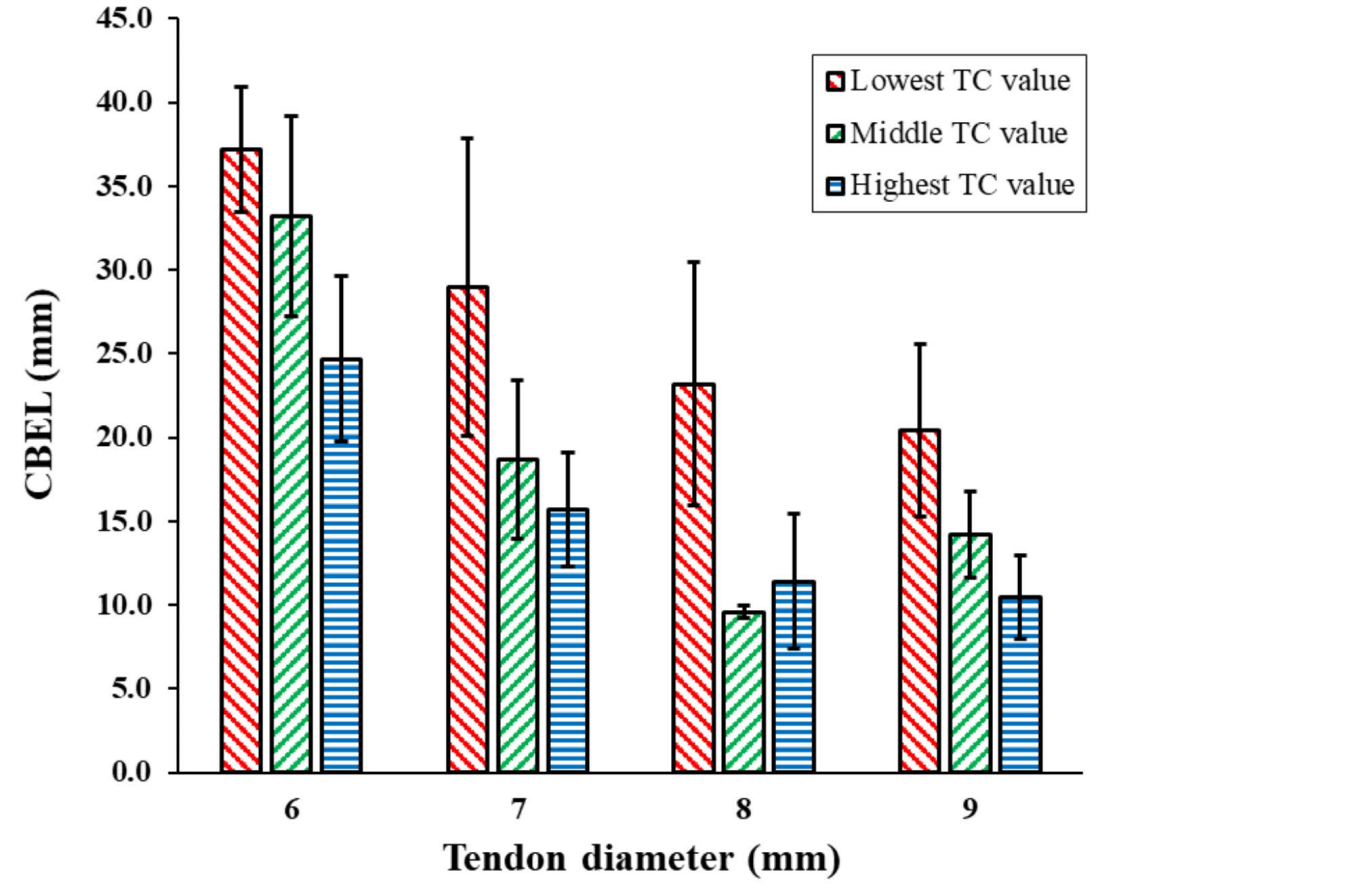
An inverse relation between CBEL and TC for each tendon’s diameter group. Error bars shown at 95% CI

Also, it was observed that the CBEL does not affect the fixation strength independently. The measurement results of CBELs for each tendon diameter group demonstrated an inverse relation between TC and CBEL (Fig. 8). Previous studies introduced TC as an effective parameter for the fixation strength of ACL reconstruction [13]. Given this inverse relationship between TC and CBEL, the latter is not an independent parameter affecting the fixation strength.

## Discussion

Primary aim of this study was to investigate the effect of the CBEL on the BASHTI fixation strength and its relationship with the cannulated drill bit and tendon diameters. Current study testified that the geometrical parameters changed the amount of the CBEL significantly. Although, the CBEL had an inverse relation with the TC, but it was not an independent parameter to consider its effect on the fixation strength. Significant differences in fixation strength were observed between TC values in each tendon diameter group (*P-value* = 0.01, 0.00006, 0.0003, and 0.012 for 6, 7, 8, and 9 mm diameters, respectively). This was in agreement with the obtained results in previous studies [13]. In this study, the effect of TC on BASHTI fixation strength was not investigated. Therefore, in each diameter group, only cases with a strength of more than 200 N were considered. According to the obtained results, the 7 mm tendon group was suggested. The higher the CBEL, the more contact area between the core bone and the tunnel surface. This might improve the healing process. Almost all of the other reconstruction methods are non-organic and there is not any bone-to-bone contact in their fixations. Due to the existence of this contact in BASHTI fixation technique, investigating the effect of geometrical parameters was a great novelty in this study.

As it was observed, during a conventional BASHTI fixation process using a hand-powered hammer with a specific insertion frequency (less than 300 beats per minute [19]), CBEL was an inverse function of TC, so it was not an independent parameter to determine the BASHTI fixation strength. Previous studies reported that the insertion frequency affects the fixation strength of BASHTI fixation [19]. This insertion frequency may change this relationship between CBEL and TC and make the CBEL an effective parameter in fixation strength. An auto-hammer with an adjustable impact frequency might improve the CBEL and TC combination, resulting in a higher fixation strength and healing process speed.

Due to the limitation of providing human cadaver bone and tendon samples in a large number (i.e., more than 60 samples), digital bovine tendon samples and Sawbones blocks were used to mimic the human bone and tendon fixation. Although the tendons used in this study were stiffer than human models, the tendon sizes were according to the sizes used in actual reconstructive ACL surgery. However, the use of the human cadaveric specimens is suggested for future study. Finally, future works should investigate the effect of insertion frequency on the CBEL and its relationship with TC and fixation strength. The core bone density was kept constant, as it was assumed that the core bone will be extracted from the same recipient source during its surgical procedure. Obtaining the core bone from other sources e.g. femoral or tibial sites with various densities may requires a further investigation to ensure the effectiveness of the described technique.

## Conclusion

The CBEL was proved to be a quality indicator influenced by the geometrical parameters, but not an independent factor on fixation strength of the BASHTI technique. The 6 mm tendons did not satisfy the 200 N fixation strength threshold. Besides, there was no significant difference in the strength of the cases that satisfied the threshold (7, 8, and 9 mm tendon groups). However, the CBEL of the 7 mm group was considerably higher than the other two groups, indicating a better healing speed for the reconstructed ACL. Consequently, the 7 mm tendon group is suggested for clinical purpose and future application of BASHTI technique.

## Data Availability

The datasets used and/or analyzed during the current study are available from the corresponding author on reasonable request.
